# EMA-FDA Parallel Scientific Advice: Optimizing Development of Medicines in the Global Age

**DOI:** 10.1007/s43441-023-00501-9

**Published:** 2023-03-04

**Authors:** Shannon Thor, Thorsten Vetter, Anabela Marcal, Sandra Kweder

**Affiliations:** 1 Europe Office, US Food and Drug Administration, Silver Spring, Maryland USA; 2 Scientific Advice Office, European Medicines Agency, Amsterdam, The Netherlands; 3 International Affairs Department, European Medicines Agency, Amsterdam, The Netherlands; 4 Office of Global Policy and Strategy, US Food and Drug Administration, Silver Spring, Maryland USA

**Keywords:** Drug development, Regulatory, EMA, FDA, Innovation

## Abstract

As medicines development continues towards a globalized approach, both the pharmaceutical industry and regulatory agencies increasingly seek opportunities to proactively engage early in product development. The parallel scientific advice program shared by the European Medicines Agency (EMA) and the US Food and Drug Administration (FDA) provides a mechanism for experts to concurrently engage in scientific discourse with sponsors on key issues during the development phase of new medicinal products (drugs, biologicals, vaccines, and advanced therapies).

## Introduction

Regulators at both the European Medicines Agency (EMA) and the US Food and Drug Administration (FDA) support and foster increasingly globalized approaches to medicines development. Covering a broad range of relevant topics in medicines development, both Agencies participate in multilateral fora such as the International Council on Harmonization (ICH), International Coalition of Medicines Regulatory Authorities (ICMRA), and the World Health Organization (WHO) to address topics such as standards setting and policy convergence at the global level. On a smaller scale, the two Agencies lead more than 30 technical working groups or “clusters” where members exchange perspectives and experiences on regulatory science topics.^1^ The cluster meetings are opportunities for regulatory experts to discuss amongst themselves challenges and difficult applications of regulatory science and policy based on the priorities of the Agencies and are not intended to serve as a forum for advising sponsors. There are situations, however, in which a developer can benefit from scientific advice on a product development program from both Agencies concurrently, and where convergent advice on the same or similar product-based scientific questions could benefit public health and facilitate patient access to needed therapies. To meet this need, EMA and FDA established a sponsor-initiated, product-specific exchange: the parallel scientific advice (PSA) program.^2^

PSA provides a mechanism for EMA and FDA experts, upon request by the applicant, to concurrently advise sponsors on scientific issues during the development of new medicinal products (drugs, biologicals, vaccines, and advanced therapies). Importantly, as part of the process the two agencies engage with each other to compare perspectives in advance of and during the actual interaction with the sponsor. This voluntary program was launched in 2005^3^ with four goals: increase dialogue between the two agencies and sponsors from the beginning of the lifecycle of a new product; provide a deeper understanding of the bases of regulatory decisions; optimize product development; and avoid unnecessary testing.

To initiate a PSA request, the applicant, herein referred to as ‘sponsor’, emails a request to each Agency.^4^ The request is expected to be brief and state the rationale for why the PSA would be beneficial, the proposed scientific questions to the Agencies, and desired goals for the meeting. If both Agencies agree to accept the request, the sponsor can move forward with preparing a full meeting package according to EMA’s Scientific Advice Working Party (SAWP) procedure schedule.^5^ A bilateral meeting between EMA and FDA takes place approximately 35 days after EMA validates the meeting package. After the bilateral meeting, preliminary feedback from each Agency is shared with the sponsor in writing. This could include preliminary responses to the sponsor’s questions or requests for the sponsor to clarify or expand a concept or proposed pathway. At approximately 65 days after validation, a trilateral meeting with the sponsor, EMA, and FDA is held. Written advice from each Agency to the sponsor follows this meeting, from EMA within ten days and within 30 days from FDA.

In 2022, we, scientists overseeing PSA at EMA and FDA, conducted a program review covering the five years from 2017 through 2021. The review included more intensive examination of a sub-cohort of submissions in calendar year 2020 to examine how well timelines were met. This paper shares the results and insights from our review and describes best practices for sponsors considering PSA.

## Methods

We independently conducted records searches in FDA and EMA files for PSA procedures requested in calendar years 2017 through 2021. The records were then merged and reviewed for accuracy and completeness. The requests were first categorized by whether they were accepted and, if not accepted, the reason. We also stratified the requests by the therapeutic area of each application’s subject product, and whether any accepted requests were later withdrawn by the sponsor. Further, we examined detailed timelines of procedural steps from the seven PSAs accepted during 2020. We selected 2020 for this sub-cohort year because when we began the records review in January 2022, the 2020 calendar year was the most recent year when all procedures had been completed and therefore had all aspects of their timelines fully characterized. For these we noted the dates of each request, acceptance, meeting package validation, and provision of the EMA Final Advice Letter.

## Results

**Figure 1:**
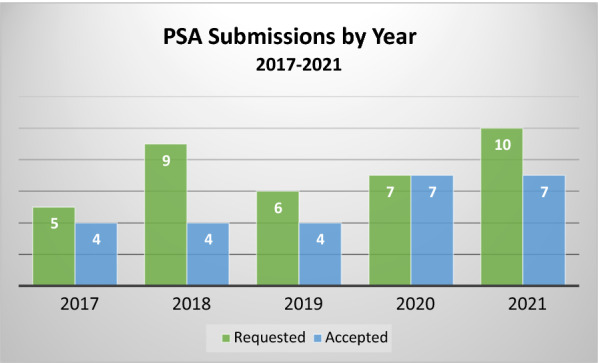
PSA requested and accepted decisions by year (2017–2021).

The 5-year review identified a total of 37 PSA requests (see Table 1). Of these, 26 (70%) were accepted to participate. Even when requests are accepted, there were times when the sponsor chose not to proceed with submitting a meeting package or formally withdrew the request. This happened four times over the 5-year period, leaving 22 completed PSA procedures, ranging from four to seven per year, as shown in Fig. 1. In no case was a request accepted and later one or both Agencies decided to discontinue the process. We note that the COVID-19 global pandemic was ongoing during the 2020 and 2021 years of this dataset. Though regulatory operations shifted to a nearly entirely virtual environment during that time, this shift did not affect the PSA program as virtual operations were already a necessary component of PSA. Further, the number of accepted requests did not decline during the pandemic years, despite both Agencies needing to shift many resources to address COVID-19 related public health needs.

**Table 1:** PSA Requests 2017–2021

Total requests	37
Accepted requests	26 (70%)
Withdrawn/package not submitted	4 (15%)
Completed procedures	22

**Figure 2:**
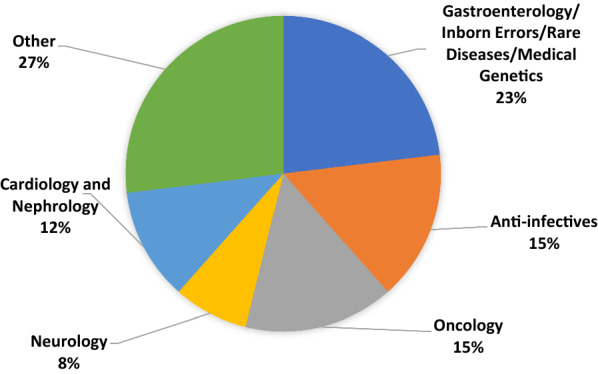
Accepted PSA requests (*N* = 26) by product category 2017–2021.

Of the accepted requests, the majority were in the therapeutic area that combines submissions for Gastroenterology, Inborn Errors of Metabolism, Rare Diseases, and Medical Genetics. We combined these into a single category for purposes of this report because during the period of our cohort FDA shifted its organizational structure and categorization of submissions. As shown in Fig. 2, Oncology, Anti-infectives, Cardiology/Nephrology, and Neurology were also areas with multiple PSA requests. Other therapeutic areas included accepted requests in Ophthalmology, Dermatology, Cardio-metabolic diseases, Pulmonology, Rheumatology, Advanced Therapies, and Hematology.

**Figure 3:**
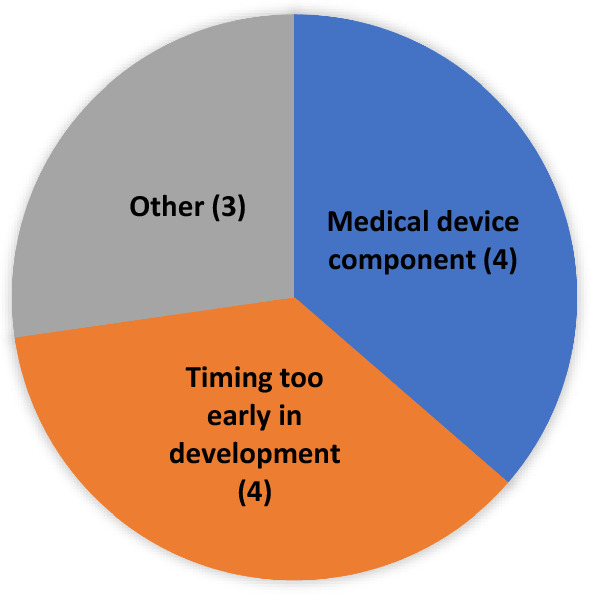
PSA requests: reason for not accepted (*N* = 11*) *At the time of these requests, EMA did not accept PSAs with a medical device component.

As previously stated, to be accepted for PSA both Agencies must agree to the request. Over the five years of our review cohort, eleven requests were not accepted (see Fig. 3). Four requests were not accepted because they were made very early in development, such as when the product had not been the subject of a pre-Investigational New Drug (pre-IND) application or IND application at FDA. Another four requests were not accepted because the request had a device component, which would not have been within EMA’s advice remit at the time (though this remit has since changed, and the EMA no longer discourages PSA submissions for products containing a device component). The other three denials involved circumstances where one or both agencies felt that PSA was not a good option for other more varied or nuanced reasons.

**Figure 4:**
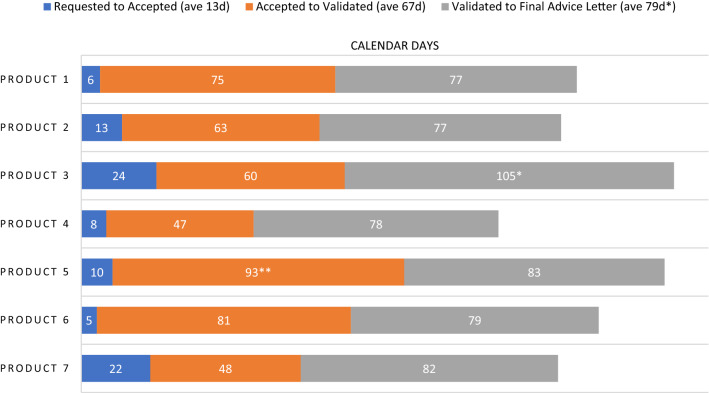
Selected Milestones for PSA Procedures in 2020. *PSA occurred over EMA SAWP August recess; not included in average, **Sponsor requested a pre-submission meeting with EMA.

Timeline data from the 2020 PSA cohort is displayed in Fig. 4. There was an average of 13 calendar days between the PSA request and the Agencies’ acceptance. Then the PSAs spent an average of 67 days in the phase of meeting package preparation and validation. Once the meeting package is validated, the Agencies begin review. During this review time multiple milestone events take place, specifically a bilateral meeting of FDA and EMA to discuss their respective reviews, followed by issuance of draft comments and further questions to the sponsor and then a trilateral meeting of the Agencies with the sponsor. A final advice letter (FAL) from EMA is issued in follow-up to the trilateral within ten days, and FDA’s meeting minutes are provided within 30 days. For six of the seven PSAs in the 2020 cohort, the average Agency review time was 79 days. There was one outlying PSA with a review time of 105 days. This PSA occurred over the period when the EMA SAWP has its annual August recess. As this is a predictable outlier that will always increase review time duration by one month, we did not include that PSA in the average for Agency review time. When we include this outlier, the average time spent under Agency review for the seven PSAs in the 2020 cohort is 83 days. Subsequent to our analysis of the 2020 cohort, we revised and published a timeline that describes each phase of PSA (Table 2).

**Table 2:** PSA timeline

Day	FDA	EMA
Anytime	Sponsor submits request for PSA to FDA and EMA Agencies decline no PSA Agencies accept Sponsor begins drafting meeting package according to SAWP procedures	
Varies		Meeting Package Submission and Validation Phase; Option for preparatory meeting with EMA according to SAWP procedures
0	EMA validates meeting package; FDA receives validated meeting package; Procedure begins	
15–25	FDA internal meeting	EMA SAWP internal discussion
30–34	FDA sends Preliminary Comments to EMA	EMA sends List of Issues to FDA
35	Bilateral FDA/EMA meeting	
65	Trilateral Sponsor/FDA/EMA meeting	
75 to 95	FDA issues final meeting minutes (30 days after trilateral)	EMA issues final advice letter (10 days after trilateral)

## Discussion

### Adding Value

For more than 15 years, PSA has been an opportunity for sponsors who are developing medicines across regulatory regions. It allows a sponsor to submit the same background and supporting material to both FDA and EMA and seek their respective advice simultaneously on the same issues. The Agencies do not commit to harmonizing advice, as each has its own regulatory frameworks. However, during a bilateral meeting they can discuss the sponsor’s questions and focus on sharing information and their perspectives in order to identify areas of convergence and divergence. In sharing their respective preliminary feedback with the sponsor in writing, including requests for further clarification and discussion, the sponsor is provided an opportunity to plan for more in-depth discussion during the subsequent trilateral meeting.

Our observation is that bringing EMA, FDA, and sponsor perspectives to a PSA trilateral setting provides a rich opportunity for all. It is common for PSA trilateral discussions to result in convergence in advice on approaches to a product’s development even though full harmonization is not always possible. And in cases of divergence, the trilateral meeting is an opportunity for the sponsor to offer proposals for how to meet both regulators’ requirements without having to explain each regulator’s perspective to the other. Even when Agencies maintain differing perspectives, an important benefit of PSA is that all parties in the process understand the reason(s) for the divergence.

### Increasing Awareness and Understanding

Typically, sponsors pursue a more traditional model of seeking advice from each Agency independently, often in series, which requires expending resources on preparing for two separate meetings where the scientific questions are often nearly identical and the burden of having to articulate one Agency’s views to the other is carried by the sponsor. When discussing PSA at a 2017 public workshop on expedited programs and regulatory harmonization, participants noted that the PSA process is not well understood by sponsors, especially the expected timelines of PSA procedures.^6^ In our observation neither our Agencies nor industry have promoted it widely and little has been written about this process. We have sought to increase awareness and understanding through public presentations,^7^ collaborating with sponsors on educational efforts,^8^ the publication of a new timetable,^9^ and this review.

Data from our 5-year review show that uptake of the PSA pathway has been limited- just four to seven procedures annually over the last five years. As described in the General Principles for PSA,^2^ PSA procedures are designed to generally correspond with the EMA’s SAWP timeline^3^ and the FDA Type B meeting^10^ timeline. Results from our 2020 cohort were consistent with these timelines. The cohort showed an average acceptance turnaround time of PSA requests at 13 calendar days; FDA Type B meeting requests are allowed up to 21 days for a response. The average review time for the cohort was 79 days, which is consistent with previously published SAWP PSA timetables predicting 75 days from the validation of the PSA meeting package to receipt of final advice.

The time from acceptance of the PSA to the validation of the meeting package varied from 47 to 93 days, with a mean of 67 days. Variation in time spent in this phase is largely within the sponsor’s control. For example, this phase may be quite short if the sponsor quickly submits a robust meeting package after their PSA request is accepted. It may be longer if the sponsor submits a deficient meeting package or requests a pre-submission meeting with EMA. The latter was the case with Product 5, shown in Fig. 4, which spent 93 days in the validation phase. Also, in some cases the sponsor delays the submission of their meeting package, for example when they are awaiting additional data.

### Looking Ahead

We have been overseeing, coordinating, and participating in the PSA program, some of us for more than a decade. Although not easy to quantify, our experience has been that once underway the outcome of the process is remarkably productive and positive for all parties. The interactions between the two regulators are critical and serve as a form of peer discussion, an opportunity to expand thinking and explore ways to address common challenges in drug development together, especially in areas where there is little experience or thorny scientific issues at hand. Products discussed under PSA are often products with no simple path forward. Therefore, EMA and FDA exploring alternative or innovative approaches together adds great value to the advice ultimately rendered to the sponsor. Such potential for value underpinned the launch of an FDA-EMA PSA pilot for complex generic products in 2021, with the hope that PSA will be a tool for optimizing global development of products for which traditional bioequivalence methods are challenging.^11^

Based on our experience and the analyses presented here, we suggest a few strategies to sponsors who are considering PSA. First, consider the timing of your request. It is strongly recommended to have begun the pre-IND or IND process at FDA on your product before requesting PSA, so that there is a baseline for reference. With the foundations and background of your product’s development plan already understood, your PSA questions can be focused on the specifics of global development that merit consideration for convergence. If timing is important to you, we further suggest that you factor into your planning the August recess of the SAWP and approximately two weeks for the Agencies’ review of your PSA request.

Second, research existing guidance on the topic to see where you can expect there is alignment across the two Agencies and where there is not. Some areas where PSA may be most appropriate are for innovative products or new scientific or regulatory concepts that have not been the subject of published guidance. Examples include advanced therapies, biosimilars, or use of novel/surrogate endpoints. Innovative manufacturing and non-clinical concepts and questions are also appropriate.

Third, consider the public health benefit of your product. PSA requires extra investment of resources from both Agencies, so the program’s focus is on products that address unmet medical needs, rare diseases, pediatric populations, or other areas of importance to patients and public health. In fact, the majority of accepted requests during the cohort period have been for rare disease therapies, pediatric populations, or advanced therapy medicinal products. Be sure to explain your product’s potential public health benefits in your request letter.

Finally, make the best possible use of the trilateral meeting. It is key to prioritize and address the issues raised in the preliminary feedback from FDA and EMA in a well-structured presentation enabling thorough and efficient discussion. This 90-min meeting is your avenue for probing both Agencies on opportunities for convergence. Hence, make sure you focus on the most critical scientific questions, and prepare proposals and rationales that address the issues noted in the preliminary feedback you received from each Agency.

## Conclusion

PSA is a longstanding EMA and FDA collaboration that continues to have strong support within both Agencies. The PSA program offers an opportunity for companies to simultaneously consult international regulators for advice on the development of important medical products, with the intent of optimizing development and deepening their understanding of regulatory decision making. Our experience has shown that the PSA program can provide timely and insightful advice on the most challenging aspects of global development. Sponsors wishing to seek PSA should consult the General Principles for Parallel Scientific Advice^2^ for further guidance.

## Footnotes

1 Tania Teixeira, Sandra L. Kweder, and Agnes Saint-Raymond. Are the European Medicines Agency, US Food and Drug Administration, and Other International Regulators Talking to Each Other?

2 GENERAL PRINCIPLES EMA-FDA PARALLEL SCIENTIFIC ADVICE. July 2021.

3 PSA Pilot Program Launch 2005.

4 Email addresses: EMAinternational@ema.europa.eu and US-FDA-EUR@fda.hhs.gov.

5 EMA Scientific Advice Working Party (SAWP).

6 Elizabeth Richardson, Gregory Daniel, David R. Joy, Sandra L. Kweder, Diane M. Maloney, Miranda J. Raggio, and Jonathan P. Jarow. Regional Approaches to Expedited Drug Development and Review: Can Regulatory Harmonization Improve Outcomes?

7 FDA Small Business and Industry Alliance Webinar: FDA-EMA Parallel Scientific Advice Program. March 2022.

8 Parallel Scientific Advice: Increasing International Dialogue Early in the Product Lifecycle. Drug Information Association Global Annual Meeting. May 2021.

9 PSA Timetable.

10 Guidance for Industry: Formal Meetings Between the FDA and Sponsors or Applicants. May 2009.

11 FDA-EMA PSA Pilot Complex Generics.

## Declarations

### Disclaimer

The views expressed in this article are the personal views of the author(s) and may not be understood or quoted as being made on behalf of or reflecting the position of the regulatory agency/agencies or organisations with which the author(s) is/are employed/affiliated.

